# Not All Academics Are Alike: First Validation of the Academics' Quality of Life at Work Scale (AQoLW)

**DOI:** 10.3389/fpsyg.2018.02408

**Published:** 2018-12-03

**Authors:** Daniela Converso, Barbara Loera, Giorgia Molinengo, Sara Viotti, Gloria Guidetti

**Affiliations:** Dipartimento di Psicologia, Università degli Studi di Torino, Turin, Italy

**Keywords:** academic work, quality of life, confirmatory factor analysis, measurment invariance, J-DR

## Abstract

**Background:** Relating to the macro-level changes and the increasing complexity of the academic system, a growing number of studies began to investigate the perceived working context impact on well-being and job satisfaction of academics. A unique duality characterizes this context: academics cannot be longer defined as stress-free, but at the same time they are still satisfied and engaged in their work. There is a need to evaluate the academic environment not only in terms of stressor and strain, but also in terms of which experiences are sources of fulfillment. The study aimed to explore psychometric properties of a new instrument (AQoLW) for assessing context-specific features of the academic work and environment that characterized academics' quality of life at work.

**Method:** A 24 item scale was deployed to academics (full, associate, and assistant professors) in a public university in the north of Italy. Items were defined to represent the main academic activities in order to measure if respondents perceived each of it as a challenging or a hindrance demand. The scale was administered online to 1,012 academics, 443 females (48.7%), mean aged 51.1 years (*SD* = 8.2). In order to test three theoretical models underling AQoLW, a training sample was randomly extracted (242 participants) and analyzed using confirmatory factor analysis (CFA). A validation sample with the remaining 668 participants was used to test the measurement invariance by role of the best model emerging from the training sample.

**Results:** Model fit demonstrate the goodness of a latent structure composed by five intercorrelated factors (CFI = 0.91, RMSEA = 0.08, SRMR = 0.07). Cronbach α of the five subscales was good, ranging from 0.76 to 0.88. The scale overtakes configural invariance, but not strong invariance by role.

**Conclusions:** The scale is able to intercept the mainly dimensions of the academic work that contribute to the quality of life of academics' staff, namely: research and public engagement, didactic work and relationships with students, career development and competition, ordinary obligations, and fund raising. AQoLW is the first tool to evaluate the academic work and its environment, identifying which activities are stressful demands and which are engaging, and promote scholars' satisfaction.

## Introduction

Over the last 20 years, changes in higher education in Western societies have resulted in an increase in research into academic working life. However, most studies have been conducted in the Anglo-American context, where the application of a market-oriented system (Clark, 1983) and new public management policies (Hood, 1995) in higher education have increased emphasis on the internationalization of the research, accountability, and university management systems of academic work (Mudrak et al., 2018). These changes, which have more recently been introduced even in European countries, have involved a complexity above the traditional academics' triple work profile (Currie, 1996; Vera et al., 2010) that is characterized by teaching, research, and institutional demands. Quantitative and qualitative studies have identified the growing cuts in research funding, increase in workload and work hours, inadequate systems of recognition and reward, longer and uncertain career paths, and work-family conflict (Doyle and Hind, 1998; Leung et al., 2000; Gillespie et al., 2001; Winefield and Jarrett, 2001; Winter and Sarros, 2002; Winefield et al., 2003; Tytherleigh et al., 2005; Houston et al., 2006; Kinman and Jones, 2008; Bentley et al., 2013; Shin and Jung, 2014) as some of the main sources of stress and dissatisfaction derived from the macro-level changes introduced by educational reforms.

Within this framework, existing research has often borrowed concepts and constructs from broader occupational health literature without paying real attention to the multifaceted complex of roles that emerged from the changes in the academic context. Several studies have indeed measured dimensions that are commonly recognized as antecedents of stress and job satisfaction among different professions, such as overload, job security and control, commitment, and quality of leadership (Leung et al., 2000; Tytherleigh et al., 2005; Melin et al., 2014; Santos, 2018). However, scientific research and academic teaching have been transformed from new market oriented procedures that concern concepts such as “rewarding,” “accountability,” “quality assurance,” “research products,” and “centers and departments of excellence” (Borrelli and Stazio, 2018). Accordingly, it is necessary to emphasize on the many competing roles and demands that have emerged within the academic field, such as the need for attracting research funding, facing performance-based evaluation processes, coping with a more competitive social climate, and tackling increased teaching and research duties. However, to date, there is no clear evidence on their value.

While it could be stated that academia can no longer be considered stress-free (Fisher, 1994), at the same time, despite being faced with multiple stressors, faculty professors are still satisfied and engaged in the primary tasks of their work, showing that the academic profession is somewhat atypical (Kinman, 2001). As highlighted, even in the presence of high workload, temporal pressure, and psychological distress, academics experience high levels of job satisfaction and intrinsic motivation, especially for teaching and research tasks (Doyle and Hind, 1998; McInnis, 2000; Bellamy et al., 2003). In this vein, a recent qualitative study (Darabi et al., 2016) investigated both negative and positive aspects of the academic profession. They reported that, while increased number of students, administrative and bureaucratic requests, and cutting of funds are the main sources of stress, several positive elements also tend to emerge. Among these, teaching, transmission of knowledge, conduct of scientific research, and autonomy maintained in the management of work were recognized as the main sources of satisfaction. Thus, the academic context is characterized by a unique duality (Kinman, 2001).

To account for this complexity, there is a need to evaluate the academic environment not only in terms of the relationship between stressor and strain, but also in terms of what academics experience as sources of satisfaction and fulfillment. Consistent with this perspective, is the Job-Demand-Resource (JD-R) model (Demerouti et al., 2001), which has further specified a means of defining job demands in their balance between job hindrances and job challenges (Van den Broeck et al., 2010). This differentiation, as highlighted from past empirical findings, leads to the conclusion that the challenging aspect of a job could simultaneously exert a role of energy-depletion and positive stimulation, which could lead to positive health and work-related outcomes, whereas hindrances simply lead to energy-depletion, which is associated with negative outcomes and ill-health (Lepine et al., 2005; Podsakoff et al., 2007). Using this framework, we can overcome some of the limitations inherent in the conceptualization of academic work characteristics as sources of stress only, also allowing the identification of challenging or stimulating elements of the work, thus orienting the development of future university management policies and practices.

To overcome the gap inherent the conceptualization and measurement of the academic working context, the present study aimed to propose a first validation of a new multidimensional instrument—Academics' Quality of Life at Work (AQoLW)—tailored to assess the increasingly demanding environment and to understand if academics perceive several dimensions related to their work as challenges or as hindrance demands.

Briefly, we had two specific aims. The first was to test the dimensions of the AQoLW scale through a confirmatory factor analysis (CFA). The second was to determine, through multisampling analysis, the invariance of the confirmatory factor model between different academic positions in Italian academia. Furthermore, considering the differentiation proposed by the JD-R model between challenges and hindrance demands (Van den Broeck et al., 2010), and to evaluate how academics perceive the context-specific characteristics evaluated by the AQoLW, we examined their correlations with occupational health-related outcomes (work engagement, emotional exhaustion, cynicism, and workaholism).

## Materials and methods

The AQoLW was developed in two steps. The first was a qualitative research phase for item generation and development, conducted by interviewing a sample of teaching and research academic staff, and content validity testing by academic experts. The second quantitative step aimed to test the psychometric properties of the new scale, such as internal consistency, construct validity, and measurement invariance, in a sample of full-time academics (Full Professors, Associate Professors, and Assistant Professors).

### Development of the item pool for the AQoLW

#### Procedure and participants

Results from a preliminary qualitative phase guided the conceptualization of the items. In-depth, face-to-face, individuals' interviews were conducted by a researcher of the Department of Psychology, between June and July of 2016, with 20 participants including 8 full professors (5 of which were head of department), 7 associate professors, and 5 assistant professors of the university where the study was conducted. A convenience sample was recruited based on different macro-scientific areas: 9 from social and economic sciences; 5 from computer, physical, and mathematical sciences; and 6 from bio-medical sciences. Majority of the participants were men (12 men and 8 women).

Topics that guided the interview were based on the literature review, and they aimed to explore the academics' perceptions about the nature of their work environment. Specifically, based on the differentiation between teaching, research, and institutional work that traditionally have defined the roles within the academia, the interviews aimed to capture (1) how the teaching and research staff described and managed these interrelated functions, and (2) what elements did they perceive as stressful or as a source of satisfaction and engagement. The interviews lasted for an average of 1 h and 30 min.

The data were analyzed using qualitative analysis based on the Grounded Theory Approach (Strauss and Corbin, 1998). By the means of this method, it was possible to evidence new emerging categories from the data, which were in turn used to develop the contents of the AQoLW. The categories that emerged from the analysis allowed us to highlight how the themes used as stimuli during the interviews, namely teaching, research, and institutional work, were enriched by new categories and meanings. Four categories emerged from the qualitative phase that guided the item development process. Regarding research work, the emerging themes pertained to public engagement activities (1) and the performance-based process of research career evaluation and competition (2). A third dimension of teaching work included aspects regarding didactic work and relationships with students (3). The fourth dimension pertained to ordinary administrative and institutional work (4). Finally, a fifth dimension, strongly related to the topic of fund raising (5), which the literature underlines as one of the most salient elements in contemporary academic life (Gillespie et al., 2001; Winefield et al., 2003; Darabi et al., 2016).

A pool of 30 items was then developed to highlight these five dimensions. The initial pool of items was evaluated by four experts in psychometrics and psychosocial research methodology, including a full professor, two associate professors, and an assistant professor of the Department of Psychology, who worked independently. For each item, the experts evaluated the clarity of wording and expression, face validity, and content validity of the construct being measured. Moreover, they judged the item response scale usability. It is important to note that, as our intent was not to measure the academic working context as stressful *per* se, but to understand if academics perceive working characteristics as challenges or as negative threatening factors (or hindrance demands), a bipolar Likert scale ranging from “Negative-stressful stimulus” to “Positive-rewarding stimulus” was used. Based on the experts' suggestions, some items were modified and 24 items were retained. The latest version of the tool was then presented to each expert independently, and was unanimously approved.

### Quantitative data collection

#### Procedure and participants

Data were collected in February and March 2017, as a part of a research program that aimed to assess work life quality within a large public higher education institution. Data used in the present study were collected from teaching and research staff, including full professors (FP), associate professors (AP), and assistant professors (AsP).

A self-report questionnaire was administered through an online survey, to the entire teaching and research staff population of the institution, which comprised 1944 persons at the time of the research. In total, 1,012 (52%) questionnaires were completed. Of these, only the full-time academic staff's questionnaires were considered for the present study. In total 910 (89.9%) questionnaires were considered valid for the analyses. The final sample included 443 females (48.7%) and 467 males (51.3%). Their mean age was 51.1 years (*SD* = 8.2 years). Regarding academic roles, 305 were AsP (33.5%), 406 were AP (44.6%) and 199 were FP (21.9%).

To test the hypothesis on the underlying dimensions of the AQoLW, a training sample comprising about the 25% of the participants was extracted randomly. A validation sample with the remaining participants was used to test the measurement invariance of the best theoretical model emerging from the training sample analyses. The size of the two samples was decided considering the requirement of at least 200 participants for each academic role in the validation sample to test for measurement invariance by academics' status. Table 1 describes the socio-demographic characteristics of the entire sample compared to the training (*N* = 242) and validation samples (*N* = 668).

**Table 1:** Study samples description: gender, age, and academic rank.

	**Training sample**	**Validation sample**	**Total**
N	242	668	910
Female	113 (46.7%)	330 (49.4%)	443 (48.7%)
Mean age (SD)	50.7 (7.8)	51.2 (8.4)	51.1 (8.2)
AsP	74 (30.6%)	231 (34.6%)	305 (33.5%)
AP	112 (43.3%)	294 (44.0%)	406 (44.6%)
FP	56 (23.1%)	143 (21.4%)	199 (21.9%)

*AsP, Assistant Professor; AP, Associate Professor; FP, Full Professor*.

#### Measures

The questionnaire included a socio-demographic section, the AQoLW, and well-established scales measuring constructs of occupational wellbeing.

Respondents were invited to rate the 24-item AQoLW using a Likert scale ranging from 1 (negative-stressful stimulus) to 7 (positive-rewarding stimulus). The scale was presented with the prompt question: “Now we ask you to evaluate some aspects of your work based on the connotation that they carry for you. Please indicate to what extent each of the aspects listed below is a negative, neutral, or positive stimulus for you.”

Moreover, the questionnaire included four health related outcome scales to assess occupational wellbeing constructs:

- Cynicism (CY) (*5 items*, α = 0.79, M = 9.27; SD = 6.05) (e.g., “I have become less enthusiastic about my work”) and emotional exhaustion (EE) (*5 items*, α = 0.85, M = 0.11.35, SD = 6.97) (e.g., “I feel emotionally drained from my work”) were measured using the corresponding subscales from the Maslach Burnout Inventory-General Survey (Schaufeli et al., 1996; Loera et al., 2014);
- Work engagement (WE) was measured using the 9-item Italian version of the Utrecht Work Engagement Scale (U-WES9) (Schaufeli et al., 2006; Balducci et al., 2010), considered as a one-dimensional scale (*9 items*, α = 0.88, M = 39.68, SD = 8.93) (e.g., At work, I feel that I am bursting with energy);
- Workaholism was measured using two subscales, working excessively (4 items, α = 0.79, M = 7.64 SD = 2.82) (e.g., “I seem to be in a hurry and racing against the clock”) and working compulsively (5 items, α = 0.85, M = 6.51, SD = 3.30) (e.g., “I feel guilty when I take time off work”), from the Italian adaptation of the Dutch Work Addiction Scale (DUWAS) (Schaufeli et al., 2009; Balducci et al., 2017).

Responses to the Burnout and Work Engagement measures were provided on a scale ranging from 0 (“Never”) to 6 (“Every day”), while those on workaholism were rated on a four-point response scale ranging from 0 (“Never”) to 3 (“Always”),

## Statistical analysis

Statistical analyses were conducted using the IBM SPSS Statistics (v. 25) software and its MATRIX language, and the Mplus 7.3 analysis program.

Data analysis was organized into the following three logically ordered phases: the first was performed using the training sample dataset, while the last two were executed on both the training and validation samples.

### Preliminary analysis on scale dimensionality: the minimum average partial velicer's test

We decided to determine the number of dimension underlying data avoiding to resort to the most popular rule of thumb in exploratory factor extraction. In fact, examining the scree-test in search of point of demarcation between major and trivial factors, and the eigenvalues greater-than-one rule may lead to underestimate or overestimate the numbers of factors (Crawford and Koopman, 1979; Streiner, 1998). Alternatively, in this study, the dimensionality of the factor structure was identified using the procedure proposed by O'Connor (2000), to perform the minimum average partial (MAP) Velicer's test. This procedure implements the MAP test to conduct a complete principal components analysis followed by the examination of k-1 (with k = numbers of observed variables) matrices of partial correlations, and directly suggests the genuine number of factors that structure an empirical correlation matrix, i.e., the step in the analyses where the average squared partial correlation was the lowest. This explorative analysis was used only to generate some insight about the dimensionality of the latent structure underlying the AQoLW.

### Confirmatory factor analysis

Since the instrument was developed following theoretical criteria and grounded information learned from the qualitative part of the study that allowed scale formulation, we had robust hypotheses on AQoLW dimensions and meaning that we decided to test directly using a CFA. Accordingly, the three theoretical alternative models were tested.

The first model, structured in three dimensions, referred to “didactic activities,” “research work,” “public engagement,” and the administrative activities underlying the other dimensions. The second model was characterized by the following four dimensions: “research and public engagement linked to research,” “didactic work and relationships with students,” “career development and competition,” and “ordinary administration/institutional and bureaucratic obligations.” The first dimension comprises activities that characterize research work through the development of a research network, work to keep oneself up to date in one's research field, and dissemination of research results. The second dimension concerns managing didactic tasks and interacting with students. The third dimension comprises the evaluative component that defines a career path within the academic context. Finally, the fourth dimension takes into consideration institutional or administrative requests posed by one's work.

Considering the increasing importance of funding in the academic context, the third model included “research and public engagement linked to research,” “didactic work and relationships with students,” “career development and competition,” “ordinary administration/institutional and bureaucratic obligations,” and “fund raising.”

Because the variables show a marked violation of normal multivariate distributions (Mardia's coefficient = 728.582, *p* < 0.000) and because the sample was large enough, maximum likelihood estimation with robust standard errors (MLR) was used for estimation (Muthén and Muthén, 2004).

The model evaluation and comparison were conducted using both incremental and absolute fit indices. We considered the models acceptable if the following criteria were satisfied: Root Mean Square Error of Approximation (RMSEA) < 0.08 (Bentler, 1990), Comparative Fit Index (CFI) > 0.90, and the Standardized Root Mean Square Residual (SRMR) < 0.08 (Hu and Bentler, 1995, 1998). In addition, the Consistent Akaike Information Criterion (CAIC) and Expected Cross-Validation Index (ECVI) to compare non-nested models (Akaike, 1987; Browne and Cudeck, 1989), and the Satorra and Bentler scaled difference (SB-Diff) to test the differences between nested models (Satorra and Bentler, 2001; Bryant and Satorra, 2012) were used.

### Multisample analysis

Multisample analyses were performed according to the procedure recommended by Reise et al. (1993). The following four hypotheses were tested: (1) the number of latent factors and the pattern of loadings are equivalent in the group; (2) the loading values are the same, (3) both factor covariance and loading values are equivalent, and (4) both the unique variances and loading values are equivalent.

In structural equation modeling terms, the four models tested were as follows:

1. The theoretical model in the groups, without imposing intergroup equivalence restrictions (baseline M1 model);
2. A model in which all the lambda matrix (Λ) coefficients are restricted to the same in all groups (M2);
3. A model in which, in addition to loadings, the error variance-covariance theta matrix (Θ) coefficients are restricted to being invariant between the groups (M3a);
4. A model in which, in addition to loadings, the latent factor covariance phi matrix (Φ) coefficients are equivalent in the groups (M3b).

The invariance hypothesis was accepted if the difference between the χ2 values of the M2, M3a, M3b models, and M1 was not significant for a number of degrees of freedom equal to the difference between the degrees of freedom in the two models (Reise et al., 1993).

### Correlation dimensions of the AQoLW and occupational health-related variables

The correlations between the dimensions of the AQoLW and occupational health-related variables were analyzed using the Pearson's coefficient on the validation sample.

## Results

### Item functioning and internal consistency in the training and validation samples

Table 2 shows the means, standard deviations, skewness (S), and kurtosis (K) for all the items in the two sub-samples (training and validation). It should be highlighted that activities that were perceived as positive, neutral, or negative were identical between the two samples. Specifically, Item 1, 4, 9, 12, 13, 14, 21, 22, and 24 were rated as neutral (with means ranging from 4 to 4.9), while Item 3, 6, 19, 20, and 23 were perceived as negative (with means ranging from 1 to 3.9). All the remaining items had a mean score above 4.9, and were perceived as positive stimuli. Additionally, the form of the distributions of the items was comparable in the two subsets of data, as higher marked non-normality (in terms of S and K) was found on the same items (Item 2, 3, 10, 11, 16, 16, and 24).

**Table 2:** Model specification.

		**M3**	**M4**	**M5**
1	Partecipare a commissioni o gruppi di lavoro dipartimentali [Participate in departmental commissions or working groups]	^*^	^*^	OA
2	Aggiornarmi rispetto agli sviluppi nel mio campo [Updating about developments in my field]	R and PE	R and PE	R and PE
3	Svolgere attività di tipo amministrativo [Doing administrative activities]	^*^	^*^	OA
4	Correggere compiti, esoneri e relazioni [Correcting students' exams, reports and thesis]	DW	DW	DW
5	Preparare le lezioni [Preparing academic lessons]	DW	DW	DW
6	Scrivere lettere e e-mail, aggiornare l'agenda [Writing letters, e-mail and updating the agenda]	^*^	^*^	OA
7	Partecipare a peer review in qualità di revisore [Participate in peer review as a reviewer]	R and PE	R and PE	R and PE
8	Fare parte di comitati editoriali o della organizzazione di convegni scientifici [Be part of editorial committees or the organization of scientific meetings]	R and PE	R and PE	R and PE
9	Svolgere attività di terza missione [Performing third stream activities]	R and PE	R and PE	R and PE
10	Partecipare a conferenze, convegni e meeting [Attending conferences, and meetings]	R and PE	R and PE	R and PE
11	Sviluppare e intrattenere collaborazioni di ricerca a livello nazionale e internazionale [Developing and maintaining research collaborations at national and international level]	R and PE	R and PE	R and PE
12	Reperire fondi necessari allo svolgimento di progetti di ricerca [Raising funds to carry out research projects]	FR	FR	FR
13	Partecipare a bandi di ricerca [Participate to research calls]	FR	FR	FR
14	Valutare la performance degli studenti [Assessing students' performance]	DW	DW	DW
15	Tenere lezioni frontali [Holding lectures]	DW	DW	DW
16	Seguire tesisti [Coordinating students's degree thesis]	DW	DW	DW
17	Seguire tirocinanti e stagisti [Coordinating trainees and apprentices]	DW	DW	DW
18	Svolgere attività di tutoring/mentoring nei confronti di dottorandi e altri giovani ricercatori [Performing tutoring / mentoring activities for PhD students and other young researchers]	DW	DW	DW
19	Competere con i miei colleghi [Compete with my colleagues]	R and PE	CC	CC
20	Partecipare ai concorsi per abilitazione [Participate in competitions for scientific qualification]	R and PE	CC	CC
21	Essere valutato sull'attività scientifica [Be evaluated on scientific activity]	R and PE	CC	CC
22	Essere valutato sull'attività didattica [Be evaluated on didactic activity]	R and PE	CC	CC
23	Seguire le procedure relative alla valutazione di ricerca e didattica [Following the procedures related to the research and teaching evaluation]	R and PE	CC	CC
24	Fare ricerca e didattica in modo funzionale alla carriera [Doing research and didactic activities functional to the career development]	R and PE	CC	CC

*DW, didactic work and relationship with students; R and PE, research and public engagement; OA, ordinary administration; CC, career development and competition; FR, fund raising*.

* *items 1, 3, and 6 are loaded on all factors, as background activities*.

Internal consistency was tested using the Cronbach's alpha, which was above satisfactory levels in both the sub-sets of data (Training sample: α = 0.871; Validation sample: α = 0.873).

### Exploratory factor analysis: scale dimensionality by the minimum average partial velicer's test

The MAP Velicer's test performed on the training sample demonstrated that the number of latent dimensions underlying the AQoLW scale was comprised between 3 and 5. The algorithm implemented two test formulas, and according to the original one (Velicer, 1976), there were 3 latent dimensions, while using the more recent (O'Connor, 2000) and revised formula, there were 5 latent dimensions. In this latest case, the smallest average squared partial correlation was 0.0196, and the smallest average 4th power partial correlation was 0.0013, i.e., there was a negligible quantity of residual variance after the extraction of five dimensions.

### Confirmatory factor analysis

The three theoretical models specified above were tested in the training sample (*N* = 242). As shown in Table 3, the five-interrelated-factors model had better fit indices than the other two did.

**Table 3:** Items description (Mean, Standard deviation, Skewness and Kurtosis).

**Item**		**Training sample**				**Validation sample**			
		***M***	***SD***	***S***	***K***	***M***	***SD***	***S***	***K***
1	Partecipare a commissioni o gruppi di lavoro dipartimentali [Participate in departmental commissions or working groups]	3.9	1.6	−0.2	−0.4	4.0	1.6	−0.2	−0.5
2	Aggiornarmi rispetto agli sviluppi nel mio campo [Updating about developments in my field]	6.4	0.9	−2.0	5.4	6.4	0.8	−1.8	4.2
3	Svolgere attività di tipo amministrativo [Doing administrative activities]	2.1	1.3	1.1	0.9	2.1	1.3	1.1	1.0
4	Correggere compiti. esoneri e relazioni [Correcting students' exams. reports and thesis]	4.1	1.3	−0.1	−0.1	4.2	1.4	0.0	−0.3
5	Preparare le lezioni [Preparing academic lessons]	5.7	1.1	−0.7	0.1	5.7	1.2	−0.8	0.8
6	Scrivere lettere e e-mail. aggiornare l'agenda [Writing letters, e-mail and updating the agenda]	3.9	1.3	0.0	−0.1	3.9	1.3	0.0	0.2
7	Partecipare a peer review in qualità di revisore [Participate in peer review as a reviewer]	5.1	1.4	−0.4	−0.2	5.1	1.4	−0.6	0.1
8	Fare parte di comitati editoriali o della organizzazione di convegni scientifici [Be part of editorial committees or the organization of scientific meetings]	5.5	1.4	−0.9	0.4	5.4	1.4	−0.8	0.4
9	Svolgere attività di terza missione [Performing third stream activities]	4.9	1.6	−0.5	0.0	4.9	1.5	−0.6	0.0
10	Partecipare a conferenze. convegni e meeting [Attending conferences. and meetings]	6.1	1.1	−1.4	1.8	6.0	1.2	−1.5	2.5
11	Sviluppare e intrattenere collaborazioni di ricerca a livello nazionale e internazionale [Developing and maintaining research collaborations at national and international level]	6.4	0.9	−1.8	3.0	6.4	1.0	−2.0	4.8
12	Reperire fondi necessari allo svolgimento di progetti di ricerca [Raising funds to carry out research projects]	4.4	1.9	−0.3	−1.0	4.2	1.8	−0.2	−1.1
13	Partecipare a bandi di ricerca [Participate to research calls]	4.5	1.8	−0.4	−0.8	4.4	1.7	−0.3	−0.8
14	Valutare la performance degli studenti [Assessing students' performance]	4.6	1.3	−0.2	−0.2	4.5	1.5	−0.3	−0.4
15	Tenere lezioni frontali [Holding lectures]	5.8	1.1	−0.9	0.3	5.8	1.1	−1.2	1.6
16	Seguire tesisti [Coordinating students's degree thesis]	5.5	1.3	−1.1	0.9	5.7	1.2	−1.2	1.8
17	Seguire tirocinanti e stagisti [Coordinating trainees and apprentices]	5.1	1.4	−0.7	0.2	5.1	1.4	−0.7	0.3
18	Svolgere attività di tutoring/mentoring nei confronti di dottorandi e altri giovani ricercatori [Performing tutoring / mentoring activities for PhD students and other young researchers]	5.9	1.2	−1.4	2.2	5.8	1.2	−1.2	1.9
19	Competere con i miei colleghi [Compete with my colleagues]	3.0	1.8	0.4	−0.8	2.9	1.7	0.5	−0.6
20	Partecipare ai concorsi per abilitazione [Participate in competitions for scientific qualification]	3.5	1.8	0.1	−0.9	3.3	1.8	0.3	−0.7
21	Essere valutato sull'attività scientifica [Be evaluated on scientific activity]	4.6	1.8	−0.5	−0.6	4.5	1.7	−0.4	−0.5
22	Essere valutato sull'attività didattica [Be evaluated on didactic activity]	4.7	1.6	−0.5	−0.3	4.7	1.6	−0.5	−0.3
23	Seguire le procedure relative alla valutazione di ricerca e didattica [Following the procedures related to the research and teaching evaluation]	3.4	1.8	0.2	−0.9	3.4	1.7	0.2	−0.8
24	Fare ricerca e didattica in modo funzionale alla carriera [Doing research and didactic activities functional to the career development]	4.4	2.0	−0.3	−1.1	4.2	2.0	−0.2	−1.1

The CFI value of 0.93 exceeded the acceptability limit, and both the RMSEA and SRMR were satisfactory. The item saturations in the respective factors were similarly high and positive (ranging from 0.32 to 0.89), and all of them were significant (*p* < 0.05). The factor correlations were also significant (*p* < 0.05), with the greatest found between F4 and F5 (*r* = 0.60), and the smallest between F2 and F3 (*r* = 0.21).

The Satorra and Bentler scaled difference between the first two nested models demonstrated that the parameter specification on four factors, instead of three, significantly improved fit (Satorra and Bentler scaled difference = 1,367, 24, *p* < 0.000), and had to be preferred.

The five-dimensional model was not nested in the fourth, but it resulted in more persuasive CAIC and ECVI; both indexes decreased substantially.

The five-dimensional model was characterized by one factor related to “research and public engagement linked to research,” a second to “didactic work and relationship with students,” a third to “fund raising,” a fourth to “career and competition,” and a fifth to “ordinary administration.”

We used the validation sample to determine if the five-factor model fit the data properly.

**Figure 1:**
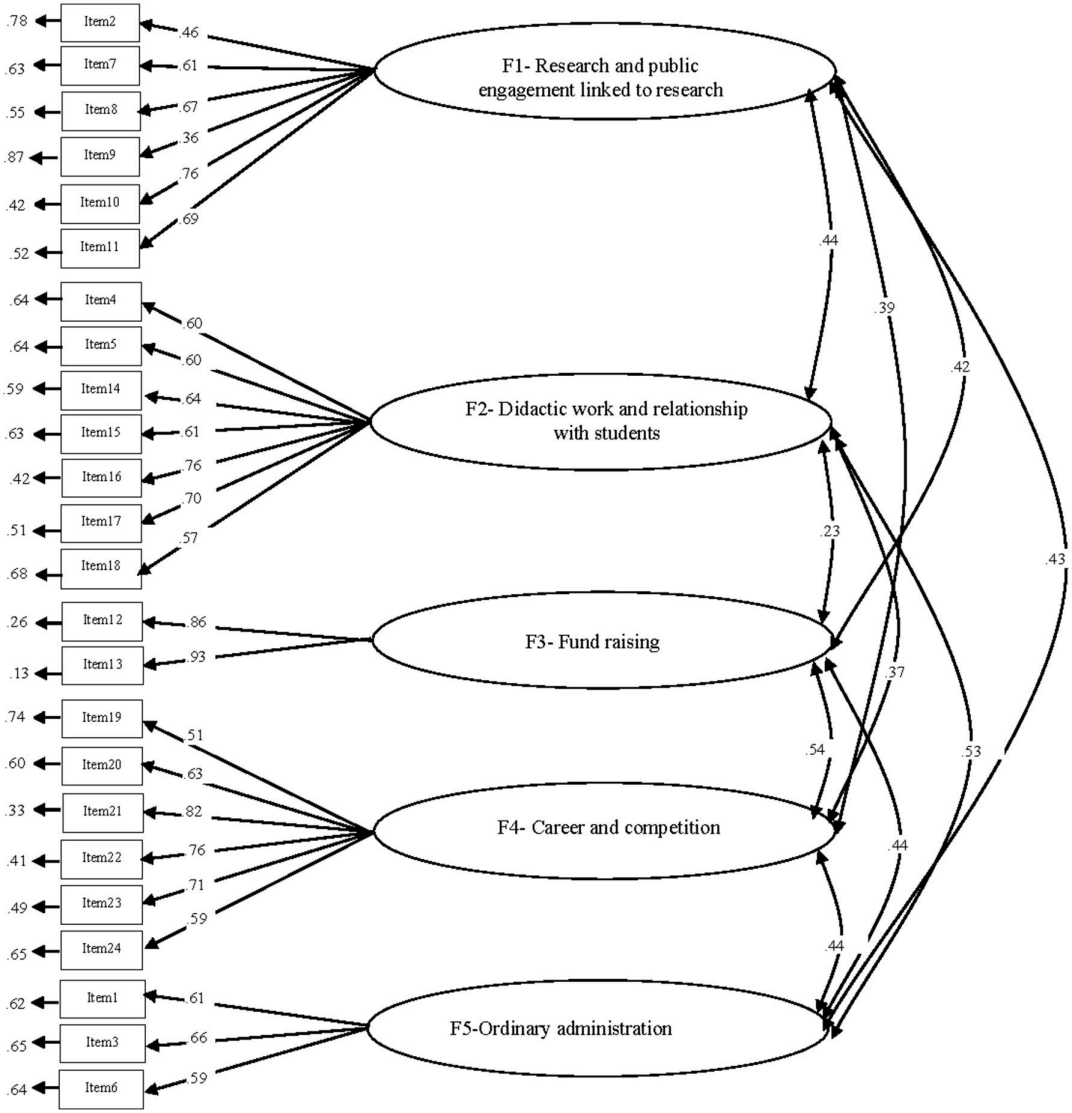
Confirmatory factor analysis on the AQoLW using data from the validation sample.

The model showed acceptable fit indexes (CFI = 0.91, RMSEA = 0.08, SRMR = 0.07, ECVI = 2.51) and obviously CAIC increased (2003.36) in function of the sample size. The item saturations were all positive and all of them were significant (*p* < 0.05), and the factor correlations were also significant (Figure 1).

The internal consistency of the five subscales, evaluated on the validation sample using the Cronbach's alpha coefficient, was good (0.76 for “research and public engagement linked to research,” 0.83 for “didactic work and relationship with students,” 0.88 for “fund raising,” and 0.83 for “career and competition”), with the exception of 0.66 for the “ordinary administration” dimension.

### Multisample analysis by role in the validation sample

The five-dimensional factor model was subjected to structural invariance analysis by academic role of the participant using the validation sample. As explained in the “Statistical Analysis” section, this process consisted of different steps.

First, the baseline model (M1) was estimated without imposing intergroup equality restrictions on parameters. The model was necessary both to check that the number of factors was the same in each group, and to test the various invariance hypotheses. The academic role invariance hypotheses tested (AsP, *N* = 231; AP, *N* = 294, FP, *N* = 143) have been presented in Table 5. The fit indices of the model, which imposed equality of loadings (M2), did not fit data and indicated that the manifest variables were indicators of the same factors in the samples and that any other types of invariance can be supposed. The invariance of error variances (M3a) and that of latent factor covariances (M3b) were not checked because the two models did not fit the data well (in terms of the fit indices CFI, RMSEA, and SRMR).

### Correlation analyses between the five dimensions of the AQoLW and occupational health-related variable

Pearson's correlations (Table 6) between all the five dimensions of AQoLW and variables related to health and well-being at work, such as work engagement, emotional exhaustion and cynicism, and workaholism, were calculated to gain some evidence of the scale's criterion validity. Since the analyses resulted in weak invariants, correlations were calculated separately by role.

All the dimensions positively and significantly correlated with work engagement and negatively with both cynicism and emotional exhaustion, except for F1 (Research and Public engagement linked to research), which was not significantly associated to emotional exhaustion but was positively and significantly correlated to workaholism (i.e., working excessively and compulsively) for AP and AsP. Finally, F5 (“ordinary administration”) was positively and significantly related to working compulsively only for AsP.

## Discussion

The present study aimed to test the psychometric properties of the Academics' Quality of Life at Work (AQoLW), a tool to assess working characteristics in the academic context. Analyses were performed to identify, through a CFA, the best theoretical model emerging from the data, using a randomized training subsample. Moreover, through a validation sample, it was tested the model that fit better from the training sample by evaluating the structure invariance among the three academic positions—Full professor (FP), Associate professor (AP) and Assistant professor (AsP) —using a multisample analysis.

Preliminary analyses showed a good internal reliability for both the training and validation samples, as Cronbach's alphas were all above 0.80. The inspection of item functioning revealed that the two sub-samples rated the items of the scale equally. As shown in Table 3, the most negative-stressful characteristics were those identified by items that referred to time-constraining activities (such as Item 3: “Doing administrative activities” and Item 6: “Writing letters, e-mails, and updating the agenda”) and activities related to the higher competition that characterizes a career path (such as Item 19: “Compete with my colleagues,” Item 20: “Participate in competitions for scientific qualification,” and Item 23: “Following the procedures related to the research and teaching evaluation”).

Activities that were either rated positively or were considered as challenging activities were identified by Item 2 (“Updating about developments in my field”), Item 5 (“Preparing academic lessons”), Item 7 (“Participate in peer review as reviewer”), Item 8 (“Be part of editorial committees or the organization of scientific meetings”), Item 10 (“Attending conferences, and meetings”), Item 11 (Developing and maintaining research collaborations at national and international level”), Item 15 (“Holding lectures”), Item 16 (“Coordinating students' degree thesis”), Item 17 (“Coordinating trainees and apprentices”), and Item 18 (“Performing tutoring/mentoring activities for PhD students and other young researchers”). A further inspection of these items revealed that the most rewarding activities for academics were those related to research and didactic activities that, consistent with past research (Darabi et al., 2016), mostly characterize the primary tasks within the university context.

“Neutral” activities were then identified by Item 1 (“Participate in departmental commissions or working groups”), Item 4 (“Correcting students' exams, reports and thesis”), Item 9 (“Performing third stream activities), Item 12 (“Raising funds to carry out research projects”), Item 13 (“Participate to research calls”), Item 14 (“Assessing students' performance”), Item 21 (“Be evaluated on scientific activity”), Item 22 (“Be evaluated on didactic activity”), and Item 24 (“Doing research and didactic activities functional to career development”). Regarding this pool of items, it should be stated that, even if they were not rated as stressful characteristics, they, at the same time, were not perceived as sources of reward or fundamental characteristics for experiencing engagement and satisfaction. Moreover, some of these activities, such as raising funds and being evaluated for teaching and research activities, represent elements introduced only by recent reforms of the Italian university system, where the present study was conducted. Evidently, it is not yet clear as to how to evaluate these new dimensions of academician' work.

The present results highlighted good psychometric properties of the AQoLW, evident both from the CFA of the training sample and from the multisample analyses conducted on the validation sample. From the CFA, a comparison of the fit indices of the three alternative models (three-, four-, and five dimensions, as shown in Table 4) indicated that the best fit was achieved from the five-dimensional model, which was confirmed from the analysis of the validation sample. The internal consistency of the scales was also satisfactory within the validation sample. As depicted in Figure 1, the first factor, composed of six items, is related to “research and public engagement linked to research,” which characterizes a broad spectrum of activities aimed at sustaining research work. Specifically, within this dimension all of the aspects describe the development and maintenance of one's research interests and networks, characterized by public engagement activities related to the dissemination of the results. The second factor, “didactic work and relationship with students,” is composed of seven items and describes all the activities that are related to the didactic dimension of academic work including, on one hand, tasks related to teaching, such as preparing for and conducting lectures, and on the other hand, more relational tasks that involve tutoring and mentoring roles. The third factor, “fund raising,” is defined by those activities that, even if part of the research task, involve different skills that are not directly linked to their own research interest. Fund raising is indeed one of the elements identified by the progressive dismantling of public funding, with its emphasis on new public management policies that require more “managerial” skills. The fourth factor, “career and competition,” identifies aspects related to the accountability system, such as being subjected to performance-based indicators and quality assurance, as well as the competitiveness that characterizes those tasks. Finally, the fifth factor, called “ordinary administration,” is composed of three items aimed at assessing elements that, even if not directly characterized as research or didactic tasks, are instrumental in the management of daily tasks.

**Table 4:** Confirmatory factor analysis in the training sample: fit indices.

**Model**	**CFI**	**RMSEA**	**SRMR**	**CAIC**	**ECVI**	**SB X^2^**	**Normal X^2^**	**DF**
Three-dimension	0.85	0.11	0.09	1399.54	4.75	899.86	1029.67	243
Four-dimension	0.92	0.08	0.07	1043.45	3.22	566.19	630.10	240
Five-dimension	0.93	0.07	0.07	1019.26	3.10	549.30	636.41	241

*CFI, Comparative Fit Index; RMSEA, Root mean square error of approximation; SRMR, Standardized Root Mean Square Residual; CAIC, Consistent Akaike Information Criterion; ECVI, Expected Cross-Validation Index; SB X^2^, Satorra and Bentler chi square*.

The multisample analysis aimed to assess if scale properties were invariant across the three academic roles that characterize the academic Italian context: full professors, associate professors, and assistant professors. The multisample analyses (Table 5) highlighted the presence of configural invariance, as the dimensionality was invariant across groups. However, no metric or scalar invariance emerged. The latter results suggest the presence of a similarity between the three sub-populations regarding the construct dimensionality, with ample divergences pertaining to the meaning of the latent factors. This fact could be interpreted in light of the role differences across the three groups, which in turn implies differences regarding the significance of each activity.

**Table 5:** Multisample analysis by academic role.

**Model**	**Matrix coefficients restricted to being equal**	**Chi-square MLR (df)**	**SBDiff**	**CFI**	**RMSEA**	**SRMR**
M1		1875.3 (723)		0.90	0.08	0.08
M2	Λ	1923.037 (763)	51.9 (100)	0.87	0.08	0.09

*Chi-square MLR, Chi-square difference using Maximum Likelihood Robust; (df), degree of freedom; SB-Diff, Satorra and Bentler scaled difference; CFI, Comparative Fit Index; RMSEA, Root mean square error of approximation; SRMR, Standardized Root Mean Square Residual*.

Finally, Table 6 shows the correlations of the five dimensions that emerged from the AQoLW with work engagement and burnout symptoms (i.e., emotional exhaustion and cynicism). Interestingly, all the dimensions significantly and positively correlated with work engagement and significantly and negatively correlated with both emotional exhaustion and cynicism, except for research and public engagement, which was not significantly associated with emotional exhaustion for AP and AsP. Another interesting finding was that, while the dimension of research and public engagement was significantly and positively related to workaholism, both with the “excessively” and “compulsively working” dimensions for AP and AsP only, only the dimension “ordinary administration” was significantly positively associated with working compulsively for AsP.

**Table 6:** Correlations among AQoLW dimensions and work engagement, burnout and workaholism by academic role.

	**Work engagement**	**Emotional exhaustion**	**Cynicism**	**Workaholism (excessively)**	**Workaholism (compulsively)**
**(FP)**					
1. RandPE	0.464^**^	−0.216^*^	−0.282^**^	0.018	0.089
2. DW	0.246^**^	−0.299^**^	−0.359^**^	−0.224^*^	−0.097
3. FR	0.204^*^	−0.192^*^	−0.286^*^	−0.171^*^	0.006
4. CC	0.285^**^	−0.299^**^	−0.296^**^	−0.279^**^	0.012
5. OA	0.262^**^	−0.228^**^	−0.387^**^	−0.188^*^	−0.072
**(AP)**					
1. RandPE	0.293^**^	−0.035	−0.179^**^	0.199^**^	0.197^**^
2. DW	0.200^**^	0.058	−0.103	−0.042	−0.010
3. FR	0.218^**^	−0.143^*^	−0.190^**^	0.067	0.081
4. CC	0.238^**^	−0.124^*^	−0.241^**^	−0.001	0.095
5. OA	0.216^**^	−0.154^**^	−0.263^**^	−0.133^*^	−0.044
**(AsP)**					
1. RandPE	0.302^**^	−0.084	−0.286^**^	0.152^*^	0.093
2. DW	0.345^**^	−0.219^**^	−0.320^**^	−0.115	−0.020
3. FR	0.195^**^	−0.084	−0.277^**^	0.061	0.050
4. CC	0.268^**^	−0.141^*^	−0.317^**^	−0.065	−0.041
5. OA	0.140^*^	−0.090	−0.228^**^	−0.199^**^	0.131^*^

*DW, didactic work and relationship with students; RandPE, research and public engagement; OA, ordinary administration; CC, career development and competition; FR, fund raising; FP, full professors; AP, associate professors; AsP, assistant professors*.

** *p < 0.001*;

* *p < 0.01*.

This correlation analysis evidenced that, within the present Italian sample, all the considered dimensions should be regarded as challenging work demands, as they probably stimulate competence and problem-solving coping strategies. Otherwise, research and public engagement linked to research constitutes a factor that could feed dysfunctional working behaviors, such as working excessively and compulsively, especially for AP and AsP, for whom the research duties are more urgent demands for their career path. Consistent with previous studies that showed a positive relationship between workaholism and poorer emotional and physical well-being (e.g., Burke, 2000), it could be hypothesized that the motivational process that sustains such activities could at the same time lead to energy-drain and more health-related problems in the long run.

In light of these latter results and the difference in job demands proposed by the JD-R model with reference to hindrances and challenges (Van den Broeck et al., 2010), it is evident that all the dimensions of the AQoLW constitute job challenges. As they positively relate to work engagement and negatively with burnout symptoms, we can hypothesize that they sustain, according to previous studies (Lepine et al., 2005; Podsakoff et al., 2007), a motivational process. However, regarding the research and public engagement dimension, it should be highlighted that, other than representing motivational sources, such work characteristics represent, in equal measure, health-related risks because they are probably fostered by workaholic behaviors.

## Conclusions and limitations

The results of the present study showed that the AQoLW is a reliable and valid instrument for the assessment of the academic working context. Differently from most of the studies and measurement tool developed for the evaluation of the academic occupational wellbeing, the AQoLW was developed to evaluate elements that specifically characterize the academic context. Engaging in research, teaching activities, and institutional tasks represent the focal activities of university professors and researchers. The AQoLW focuses on these factors, simultaneously considering the changes that these roles have undergone as a result of market-oriented reforms applied to educational systems. Activities related to fund raising, public engagement, and being subjected to evaluative processes of one's own teaching and research outcomes (all typical of new public management reforms) are indeed, to date, understudied. In light of this, the AQoLW could represent a reliable instrument to assess the multiple and competing roles that characterize today's academic working life, providing impetus for future studies in this field of research.

Moreover, given differences between educational systems across countries, this instrument could represent a valid tool to assess elements that, as highlighted from past studies (Shin and Jung, 2014), are common to most Western societies that have adopted systems based on new public policies. Future studies should therefore propose further validation that takes into consideration cross-cultural differences.

The present study has some limits. First, we cannot generalize the results, even if based on a large sample of a large Italian public university. Future studies could overcome this limit, proposing this tool to larger samples of academics that would be more representative of the Italian university system. Moreover, the absence of scalar and metric invariance regarding differences related to different academic roles should be further inspected in future studies. Finally, future studies should perform test-retest reliability, which was not possible to assess within the research design of this study.

## Ethics statement

The present study is part of a larger study on psychological well-being at work, requested by the Rector of the University of Turin, and approved by the board of administration and the administrative and academic union delegations of the mentioned university. To guarantee the scientific value of the study and respect for all norms linked to privacy and ethics, a specific board was assigned to control the research design and administration. The research conformed to the 1995 Declaration of Helsinki (as revised in Edinburgh in 2000) and all the contents of the interviews and questionnaire were previously approved by the university committee that commissioned the project (Gruppo di lavoro per la valutazione dello stress lavoro correlato e della qualità; della vita organizzativa), which confirmed if they appropriately evaluated work-related stress and quality of working life. The entire population was clearly informed about the research aims and outcomes. Voluntary participation and anonymity of data collection were ensured. In fulfilling the on-line questionnaire, participants check a box in order to give their informed consent. No treatment, including medical, invasive diagnostics, or procedures causing psychological or social discomfort was administered to the participants; therefore, no additional ethical approval was required.

## Author contributions

DC, BL, GG, GM, and SV research conception and design, acquisition of data, critical revision of the article content. BL and GM quant data analysis. GG and MG qual data analysis. BL, GG, GM, and DC interpretation of data, drafting the article.

### Conflict of interest statement

The authors declare that the research was conducted in the absence of any commercial or financial relationships that could be construed as a potential conflict of interest.
